# Influence of destructive leadership behaviors on the meaning of work and work productivity

**DOI:** 10.3389/fpsyg.2023.1295027

**Published:** 2023-12-13

**Authors:** Martin Grill

**Affiliations:** Department of Psychology, University of Gothenburg, Gothenburg, Sweden

**Keywords:** incoherent planning, assigning unnecessary tasks, ambiguous expectations, autocratic behavior, work engagement, illegitimate tasks, industrial psychology, longitudinal studies

## Abstract

This study aimed to determine the influence of destructive leadership behaviors on employees’ meaning of work and work productivity, using a longitudinal research design. Local government organizations in a municipality in Sweden were invited to participate in the study. Self-rated questionnaire data on employees’ meaning of work and work productivity was collected at four time points over a period of 18 months, and 582 employees responded to the questionnaire on one or more occasions. A 4-item Destructive Leadership Scale (DLS) was developed and used at the first time point to assess the destructive leadership behaviors of incoherent planning, assigning unnecessary tasks, ambiguous expectations, and autocratic behavior. Latent growth models were used to analyze the influence of destructive leadership on the change in employees’ meaning of work and work productivity over the 18-month period. The results show that destructive leadership has a significant negative influence on employees’ meaning of work (*β* = −0.44, *p* = 0.02) and work productivity (*β* = −0.46, *p* = 0.04). The effect sizes were greater than those identified in previous cross-sectional studies, indicating that the effects of destructive leadership may accumulate and become more important over time. Important destructive leadership behaviors include incoherent planning, assigning unnecessary tasks, ambiguous expectations, and autocratic behavior. These behaviors have a significant negative effect on employees’ meaning of work and work productivity. Proactive assessment of destructive leadership behaviors is warranted to improve future selection and training of managers.

## 1 Introduction

For organizations to survive and prosper, it is not enough for employees to be productive; employees must also perceive their work as being meaningful, important, and motivating (Pejtersen et al., 2010; Tóth-Király et al., 2023). Managers’ destructive leadership behaviors have been found to correlate negatively with employees’ job performance, engagement, and well-being, and positively with employees’ burnout, stress, health complaints, and workplace deviance (Montano et al., 2017, 2023; Li et al., 2021). However, most studies on the consequences of destructive leadership have been cross-sectional, which severely limits any claims of causality. Therefore, this study aimed to determine the influence of destructive leadership behaviors on employees’ meaning of work and work productivity using a longitudinal research design.

The two most common conceptualizations of destructive leadership behaviors are avoidant leadership (Avolio et al., 1999) and abusive leadership (Tepper, 2007). *Avoidant leadership*, which involves the absence of proactive leadership behaviors, includes avoiding decision-making and failing to take action until problems become serious (Avolio et al., 1999). *Abusive leadership*, which involves actively hostile leadership behaviors, includes invading employees’ privacy and putting them down in front of others (Tepper, 2007).

However, subtle actively destructive behaviors may also play an important role in destructive leadership. Based on interviews with local government managers, Eklöf et al. (2010) aimed to comprehensively describe the sources of stress in local government organizations, including managers’ destructive leadership. Their findings provide a stepping stone for the development of a destructive leadership conceptualization based on managers’ perspectives—a conceptualization that can provide an insightful and nuanced understanding of destructive leadership behaviors (Liao et al., 2018). Four types of destructive leadership behaviors can be extracted from the findings of Eklöf et al. (2010): *incoherent planning* (e.g., deficient planning behaviors), *assigning unnecessary tasks* (e.g., making decisions that generate unnecessary tasks), *ambiguous expectations* (e.g., communicating unclear demands), and *autocratic behavior* (e.g., ignoring others’ views).

Grill and Nielsen (2019) found managers’ incoherent planning behaviors to be potentially harmful and an important aspect of destructive leadership. They argue that a lack of or inadequate planning reduce structure and predictability at work and increase *ad hoc* decision-making; when employees face an unstructured and unpredictable work situation and are forced to adjust their work to *ad hoc* decisions from their managers, their work becomes more reactive and their own long-term planning is undermined, potentially reducing their productivity and motivation.

Assigning unnecessary tasks was recently suggested (Stein et al., 2020) to be an essential feature of destructive leadership. A systematic literature review (Ding and Kuvaas, 2022) highlighted leadership as an important antecedent of unnecessary tasks; having to perform unnecessary tasks keeps employees away from performing necessary tasks, which may reduce their productivity. Also, Stein et al. (2020) argued that assigning unnecessary tasks sends a message of disrespect and devaluation to employees, which can cause demotivation and decrease employees’ meaning of work.

Ambiguous expectations may induce inconsistencies in employees’ work roles (i.e., the contents of tasks, expectations to be met, and employee responsibilities), which may lead in turn to a deterioration of role clarity—a core psychosocial work environment factor (Burr et al., 2019). Podsakoff et al. (2006) have described how inconsistencies between managers’ antecedent and consequential leadership can generate unclear expectations and decrease employee productivity; for example, managers may prompt employees to work toward one organizational goal while the reinforcing contingencies reward performance that is in line with other goals. If it is unclear what is expected of employees, it is less likely that employees will engage in the most productive tasks. Also, unclear expectations can obscure the link between employee performance and organizational performance, preventing employees from understanding how their work contributes to meaningful organizational outcomes (Binder, 2016).

Autocratic behavior includes disregarding input from employees, which may discourage them from participating in work-related activities and reduce their motivation (Grill et al., 2023). Grill et al. (2023) argued that employees’ knowledge and experience must be considered in order for their work to be productive, and being listened to allows employees to feel that their input is valuable, important, and meaningful.

**Figure 1:**
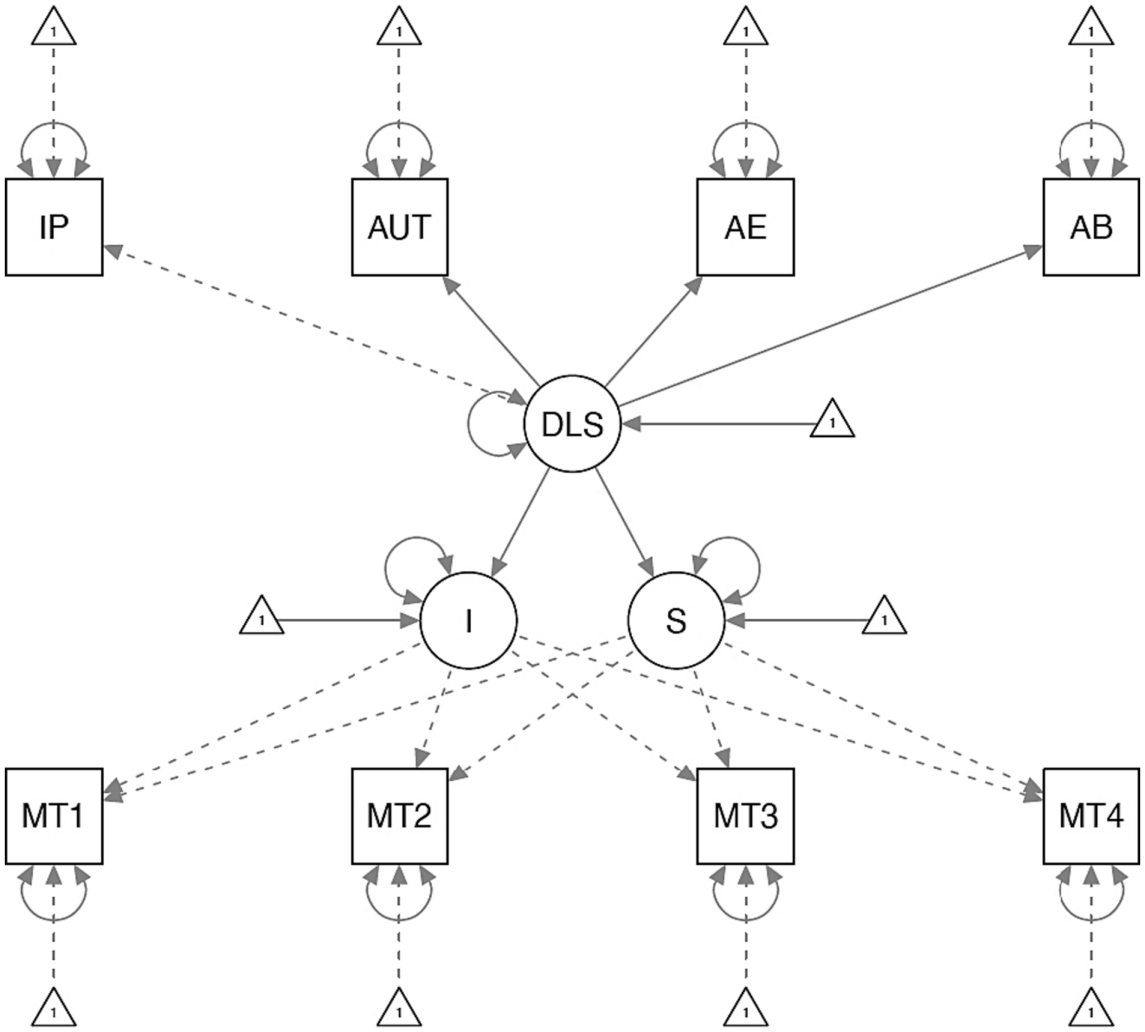
Conceptual model of how to estimate the long-term effect of destructive leadership behaviors on meaning of work. IP, Incoherent planning; AUT, Assigning unnecessary tasks; AE, Ambiguous expectations; AB, Autocratic behavior; DLS, Destructive Leadership Scale; I, Intercept in meaning of work; S, Slope in meaning of work; MT1-4, Meaning of work at the first, second, third, and fourth time point.

In Montano et al.’s (2023) meta-analysis of seven leadership constructs, destructive leadership had the second strongest (negative) correlation with followers’ positive mental health. However, correlation is only the first prerequisite for causal inference (Shadish et al., 2002). Furthermore, temporal precedence of the cause must be established. Latent growth models (LGMs; Duncan et al., 2013) permit an investigation of how the level of destructive leadership can influence subsequent changes (i.e., the slope) in meaning of work and work productivity. Figure 1 illustrates a conceptual LGM of how to estimate the effect of destructive leadership behaviors on changes over time in meaning of work and work productivity.

*Hypotheses*: The destructive leadership behaviors of incoherent planning, assigning unnecessary tasks, ambiguous expectations, and autocratic behavior are related to a decrease over time in employees’ (1) meaning of work and (2) work productivity.

## 2 Methods

### 2.1 Procedure and participants

Employees (*n* = 712) in local government organizations in a municipality in Sweden were invited to participate in the study. The participating organizations encompassed multiple types of operations, including: education; healthcare; construction and development; management and maintenance of property, water, drainage, and waste; social and emergency services; elderly care; transport; administration and economics; cultural administration; tourism; communication; and human resource management. An online questionnaire was distributed to the employees at four time points at six-month intervals: October–November 2019 (T1), April–May 2020 (T2), October–November 2020 (T3), and April–May 2021 (T4). At each time point, updated e-mail lists were collected to allow for the inclusion of newly employed individuals. In total, 582 employees (82%) responded to the questionnaire on one or more occasions (502/71% at T1, 449/65% at T2, 369/59% at T3, and 297/57% at T4). The respondents were 60% female and 74% had a university education; their average age was 44 years (SD = 11). The respondents were clustered among 72 managers. The managers were on average 47 years old (SD = 6.8); 57% were female, and 49% had participated in leadership training.

### 2.2 Measures

The predictor variable was measured at the first time point with a four-item Destructive Leadership Scale (DLS; Appendix A) based on the work of Eklöf et al. (2010). The DLS includes four types of destructive leadership behavior: incoherent planning (i.e., “How often does your manager demonstrate deficient planning behaviors?”); assigning unnecessary tasks (i.e., “How often does your manager make decisions that generate unnecessary tasks?”); ambiguous expectations (i.e., “How often does your manager express ambiguous expectations?”); and autocratic behavior (i.e., “How often does your manager ignore your views?”). The responses were recorded using a Likert-type scale ranging from 1 (never) to 5 (frequently, if not always). A confirmatory factor analysis for a one-factor solution showed adequate goodness of fit [*χ*^2^(2) = 5.94, CFI = 0.992, RMSEA = 0.069, SRMS = 0.017] and approximately equal factor loadings for all item (incoherent planning: *β* = 0.68; assigning unnecessary tasks: *β* = 0.83; ambiguous expectations: *β* = 0.64; and autocratic behavior: *β* = 0.67). McDonald’s *ω* was determined to be 0.80, and the intraclass correlation coefficient (ICC) was 0.10.

The outcome variables were measured at four time points and comprised the three-item Meaning of Work Scale developed by Pejtersen et al. (2010) (i.e., “Is your work meaningful?,” “Do you feel that your work is important?,” and “Do you feel motivated and involved in your work?”) and a three-item productivity scale adapted from von Thiele Schwarz et al. (2014), which includes three productivity dimensions: efficiency (i.e., “How would you describe your work efficiency during the last week?”), quantity (i.e., “How would you describe the quantity of the work you have done during the last week?”), and quality (i.e., “How would you describe the quality of the work you have done during the last week?”). The responses for meaning of work were recorded using a Likert-type scale ranging from 1 (to a very small extent) to 5 (to a very large extent), and the measure of productivity used a scale ranging from 1 (my worst ever) to 10 (my best ever). For meaning of work, McDonald’s *ω* was determined to be 0.85 (T1), 0.87 (T2), 0.84 (T3), and 0.85 (T4), while the ICC was 0.12 (T1), 0.12 (T2), 0.13 (T3), and 0.13 (T4). For work productivity, McDonald’s *ω* was 0.86 (T1), 0.87 (T2), 0.90 (T3), and 0.89 (T4), while the ICC was 0.07 (T1), 0.12 (T2), 0.08 (T3), and 0.07 (T4).

Time invariance was assessed following Cheung and Rensvold’s (2002) change in comparative fit index (ΔCFI) < 0.01 criteria. The meaning of work measure was found to be time-invariant: The ΔCFI was <0.01 in all stepwise comparisons of models with no constraints (CFI = 0.992), with constrained factor loadings (CFI = 0.984), and with constrained intercepts (CFI = 0.979). The work productivity measure was also found to be time-invariant: The ΔCFI was <0.01 in the comparisons of models with no constraints (CFI = 0.993), with constrained factor loadings (CFI = 0.989), and constrained intercepts (CFI = 0.986).

### 2.3 Data analysis

Two latent growth curves (Duncan et al., 2013) were modeled—one for meaning of work and one for work productivity—in R (version 2023.03.1) with the Lavaan package (version 0.6.14; Rosseel, 2012) using robust maximum likelihood (MLR) estimations of standard errors and test statistics. Mean level indexes were created for the outcome variable at each time point. The factor loadings for the slope were constrained to zero for T1, one for T2, two for T3, and three for T4. The hypotheses were tested by assessing the effects of the DLS (mean centered) on the change (i.e., slope) in meaning of work (H1) and work productivity (H1). To obtain representative parameter estimates, a design-based LGM (Wu et al., 2014) was used by including higher level control variables—that is, the managers’ age, gender, and training. Age, gender (Eagly et al., 2003), and training (Grill et al., 2023) are factors known to influence leadership performance.

With clustered data, disaggregated modeling (i.e., “multi-level analysis”) should be considered (Muthén, 1997). However, the low ICC values (0.07–0.13) indicated that most of the variation in data was on the individual level. Therefore, the variation in the respondents’ experiences of their meaning of work, their work productivity, and the destructive leadership behaviors of their managers was primarily an individual matter rather than being shared between individuals within workgroups. The low ICC values in combination with the small number of individuals in each group (*M* = 8) implies that the cluster effect was very small, or even “ignorable” (Muthén, 1997, p. 457). Hence, aggregated modeling was used. However, to handle any non-independence in data caused by the clustering, a sandwich estimator and test statistics equivalent to the T_2_^*^ test statistic of Yuan and Bentler (2000) were used to estimate cluster-corrected standard errors and test statistics robust to non-independence of observations (Muthén and Muthén, 2010, p. 533).

MLR was also used to handle missingness in data (Yuan and Bentler, 2000). Traditionally, missingness has been handled with listwise deletion, a procedure that generates “grossly inefficient estimates” (Yuan and Bentler, 2000, p. 191) and severely limits the generalizability of the results. Instead, MLR handles missingness by using all information in the data and weighting the information so that respondents who have answered at all time points contribute more to the results than respondents who have answered at fewer time points. Hence, generalizability was improved by continuously including newly employed individuals and using MLR to estimate more representative standard errors and test statistics.

However, to make sure that the results were not caused by any spurious effect of the higher level control variables or any bias introduced by respondents entering the study at later stages, sensitivity analyses were performed without any control variables and including only respondents with complete data.

## 3 Results

**Figure 2:**
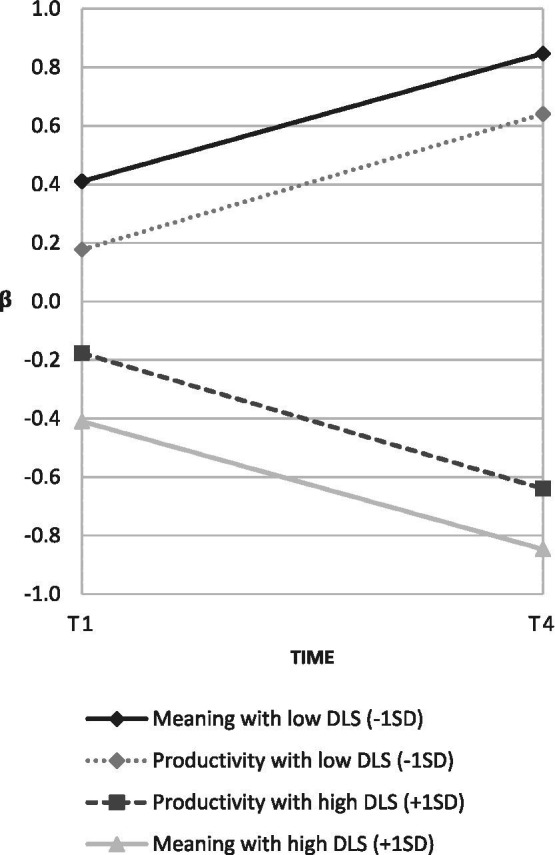
Standardized effects (β) of destructive leadership (DLS) on the level of (i.e., intercept) and change in (i.e., slope) meaning of work and work productivity.

The descriptive statistics and intercorrelations of the study variables are presented in Table 1. The results from the LGMs are outlined in Table 2 and show that destructive leadership had a significant negative effect on the change in meaning of work (*β* = −0.44, *p* = 0.02) and work productivity (*β* = −0.46, *p* = 0.04). These effects imply that employees with managers exhibiting a high rate of destructive leadership behaviors experienced a deterioration in meaning of work and work productivity. The effects are illustrated in Figure 2. Of the control variables, training had a significant positive effect on the change in work productivity (*β* = 0.29, *p* = 0.04), while age and gender did not. None of the control variables had any significant effect on the change in meaning of work. The sensitivity analyses—without control variables and including only respondents with complete data (*n* = 219)—confirmed that destructive leadership has a significant negative effect on the change in employees’ meaning of work (*β* = −0.65, *p* < 0.01) and work productivity (*β* = −0.98, *p* < 0.01).

**Table 1:** Descriptive statistics and correlations for study variables.

Scale	*M*	*σ* ^2^	1	2	3	4	5	6	7	8
1. DLS	2.18	0.48								
2. Meaning T1	4.28	0.41	−0.31^*^							
3. Meaning T2	4.24	0.43	−0.45^*^	0.74^*^						
4. Meaning T3	4.25	0.44	−0.39^*^	0.70^*^	0.78^*^					
5. Meaning T4	4.17	0.60	−0.39^*^	0.72^*^	0.73^*^	0.76^*^				
6. Prod. T1	7.08	1.79	−0.13^*^	0.30^*^	0.23^*^	0.25^*^	0.21^*^			
7. Prod. T2	7.05	2.33	−0.23^*^	0.29^*^	0.38^*^	0.36^*^	0.31^*^	0.53^*^		
8. Prod. T3	7.12	1.92	−0.26^*^	0.24^*^	0.28^*^	0.38^*^	0.30^*^	0.49^*^	0.63^*^	
9. Prod. T4	6.94	2.40	−0.32^*^	0.22^*^	0.31^*^	0.21^*^	0.28^*^	0.47^*^	0.52^*^	0.58^*^

A confirmatory factor analysis—with the intercepts constrained to zero and the factor loadings to one for the first item in each scale—was used to provide descriptive statistics and correlations between the study variables [*χ^2^*(278) = 466.58, *n* = 582, CFI = 0.970, RMSEA = 0.046, SRMS = 0.051]. DLS, Destructive Leadership Scale; Meaning, Meaning of work; Prod., Work productivity; σ^2^, variance. ^*^p < 0.05.

**Table 2:** Results from the LGMs provide estimates for the influence of destructive leadership (DLS) on meaning of work and work productivity.

	Meaning of work					Work productivity				
	Estimate	*SE*	*z*	*p*	*β*	Estimate	*SE*	*z*	*p*	*β*
Intercept	4.167	0.083	50.229	<0.001	7.236^*^	7.088	0.125	56.787	<0.001	7.661^*^
Slope	−0.058	0.021	−2.740	0.006	−0.579^*^	−0.074	0.047	−1.550	0.121	−0.399
**Intercept predictors**										
Managers’ DLS^a^	−0.345	0.059	−5.821	<0.001	−0.410^*^	−0.239	0.107	−2.238	0.025	−0.177*
Managers’ gender^b^	−0.075	0.077	−0.972	0.331	0.065	0.341	0.134	2.549	0.011	0.182^*^
Managers’ age^c^	−0.002	0.025	−0.425	0.671	−0.024	−0.005	0.010	−0.456	0.648	−0.033
Managers’ training^d^	0.025	0.072	0.341	0.733	0.021	−0.032	0.136	−0.235	0.814	−0.077
**Slope predictors**										
Managers’ DLS^a^	−0.063	0.028	−2.254	0.024	−0.437^*^	−0.125	0.062	−2.017	0.044	−0.463^*^
Managers’ gender^b^	0.020	0.024	0.840	0.401	0.099	−0.023	0.052	−0.451	0.652	−0.063
Managers’ age^c^	−0.004	0.002	−1.950	0.051	−0.264	0.001	0.004	0.341	0.733	0.053
Managers’ training^d^	0.012	0.021	0.575	0.331	0.061	0.106	0.051	2.052	0.040	0.286^*^
**Goodness of fit**										
Scaled *χ^2^* (*df*)	65.100 (42)						59.765 (42)					
Robust CFI	0.973					0.985				
Robust RMSEA	0.050					0.029				
SRMS	0.039					0.041				

*n* = 582. ^a^DLS, Destructive Leadership Scale (mean centered). ^b^Centered at male. ^c^Mean centered years. ^d^Centered at no training. ^*^p < 0.05.

## 4 Discussion

This study aimed to determine the influence of destructive leadership behaviors on employees’ meaning of work and work productivity, using a longitudinal research design. The results indicate that the destructive leadership behaviors of incoherent planning, assigning unnecessary tasks, ambiguous expectations, and autocratic behavior had a significant negative effect on employees’ meaning of work and work productivity over an 18-month period. Employees with managers who demonstrate higher levels of destructive leadership behavior are more likely to experience a decrease in their meaning of work and work productivity than employees with managers who demonstrate lower levels of destructive leadership behaviors.

This finding supports cross-sectional research on the impacts of destructive leadership behaviors (Montano et al., 2017, 2023; Li et al., 2021). Moreover, the effect sizes identified in the present study were larger than those identified in previous cross-sectional studies, indicating that destructive leadership behavior may be more important than previously acknowledged. Similar results were found in Li et al.’s (2021) meta-analysis on the relationship between leadership and employee engagement: Longitudinal studies on destructive leadership report greater effect sizes than cross-sectional studies. A comparison of the effect sizes found in the present study with the effect sizes for different types of beneficial leadership behaviors (e.g., Li et al., 2021) indicates that preventing destructive leadership behaviors may be of more importance than promoting positive leadership behaviors.

This study shows that subtle actively destructive behaviors seem to be of particular importance and hence merit further attention in future research and practice. Subtle destructive leadership behaviors may also be more common in workplaces than flagrantly abusive leadership behaviors and thus pose a larger problem in organizations. The DLS developed for this study is available for future research and practical applications.

### 4.1 Limitations

Naturalistic randomized controlled trials (RCTs) may be the best way to determine causality (Shadish et al., 2002). In this study, two of the three grounds for causality (i.e., correlation and temporal precedence) were established. However, the final ground (i.e., ruling out other explanations)—which is elegantly handled in RCTs—was left with limited attention, since only three possible confounders were included in the analysis. In future research, random allocation of destructive leadership may be cautiously considered, so that more confounders can be adjusted for and stronger causal inferences can be provided.

The present study coincided with the Covid-19 pandemic, an event that has had a severe impact on all parts of society. It is unknown to what extent the results of this study can be generalized to post-Covid societies. The impact of destructive leadership may be smaller or greater during times of crisis; hence, replication in times less affected by crisis is needed to assess the generalizability of the results.

### 4.2 Conclusion

The findings of this study show that the destructive leadership behaviors of incoherent planning, assigning unnecessary tasks, ambiguous expectations, and autocratic behavior have a significant negative effect on employees’ meaning of work and work productivity. Organizations may need to focus more on these gloomier aspects of managers’ behavioral repertoire in the selection and training of managers—and in other strategic decisions related to leadership and talent management—in order to survive and prosper in the future.

## Data availability statement

The raw data supporting the conclusions of this article will be made available by the author without undue reservation.

## Ethics statement

The studies involving humans were approved by the Swedish Ethical Review Authority Dnr 1060-18/2019-00590. The studies were conducted in accordance with the local legislation and institutional requirements. The participants provided their written informed consent to participate in this study.

## Author contributions

MG: Conceptualization, Formal analysis, Funding acquisition, Investigation, Methodology, Project administration, Writing – original draft, Writing – review & editing.

## Figures and Tables

**Table:** 

		Never	Once in a while	Sometimes	Fairly often	Frequently, if not always
1.	How often does your manager demonstrate deficient planning behaviors?	☐	☐	☐	☐	☐
2.	How often does your manager make decisions that generate unnecessary tasks?	☐	☐	☐	☐	☐
3.	How often does your manager express ambiguous expectations?	☐	☐	☐	☐	☐
4.	How often does your manager ignore your views?	☐	☐	☐	☐	☐

## Appendix A

The Destructive Leadership Scale (DLS). Rate how frequently your manager displays the following behaviors
